# POPs and Pubertal Timing: Evidence of Delayed Development

**DOI:** 10.1289/ehp.123-A266

**Published:** 2015-10-01

**Authors:** Nate Seltenrich

**Affiliations:** Nate Seltenrich covers science and the environment from Petaluma, CA. His work has appeared in *High Country News*, *Sierra*, *Yale Environment 360*, *Earth Island Journal*, and other regional and national publications.

Endocrine disruptors have been eyed as potential drivers of a steady trend toward earlier puberty among girls worldwide in recent decades, particularly with regard to breast development.^1^^,^^2^^,^^3^^,^^4^ However, when the authors of a study in this issue of *EHP* evaluated serum levels of three common classes of hormonally active persistent organic pollutants (POPs) in relation to the timing of pubertal onset in girls, they found, contrary to initial hypotheses, that higher exposures were associated with later puberty, not earlier.^5^

A small but growing body of literature suggests a complicated relationship between contaminants and pubertal development; factors such as stress, diet, and exercise also play an important role, says lead author Gayle Windham, a research scientist at the California Department of Public Health. Both earlier and later puberty have implications for psychosocial development,^6^ while early onset increases the risk of a range of physical health outcomes associated with prolonged exposure to estrogen,^7^ including breast cancer.^8^

**Figure:**
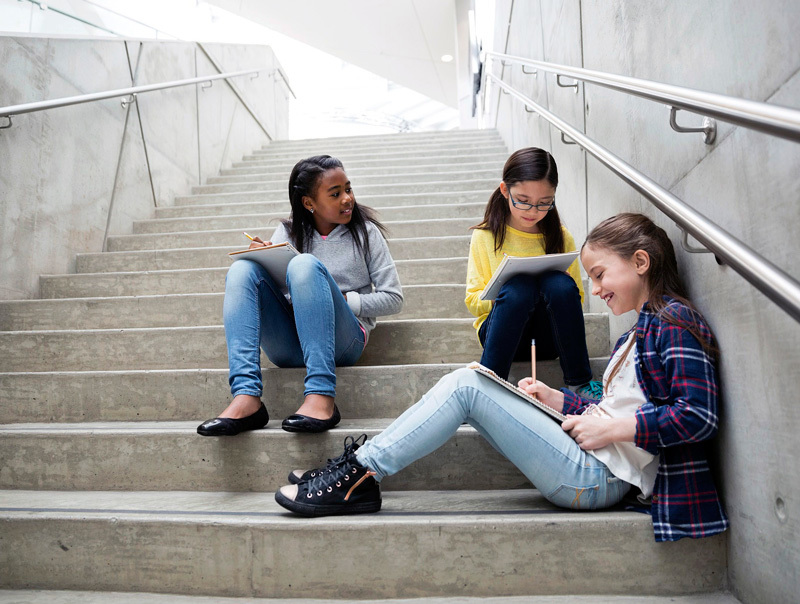
Chemical exposures, along with stress, diet, and exercise, play a complicated role in the timing of pubertal development. © Hero Images/Corbis

The current study involved a cohort of more than 600 ethnically diverse girls who were enrolled in the Puberty Study of the NIH-funded Breast Cancer and the Environment Research Program.^9^ The girls were enrolled between 2004 and 2007 when they were 6, 7, or 8 years old. Quartiles of exposure to polybrominated diphenyl ethers (PBDEs), polychlorinated biphenyls (PCBs), and organochlorine pesticides were determined based on blood samples collected at enrollment. Pubertal development was subsequently monitored at up to seven annual clinic visits. Across the three classes of chemicals, breast development occurred a median of 6–11 months later among the most highly exposed girls versus those least exposed.^5^

While epidemiological evidence for the role of environmental exposures in pubertal timing remains mixed and inconclusive, researchers have reported an association between obesity and earlier puberty in girls.^10^^,^^11^ This may be due at least in part to the fact that fat cells produce estrogen.^12^ But the relationship is not one-directional, as more physically mature girls are also likely to have more body fat.^13^

When it comes to exposures to lipophilic chemicals such as those studied here, fat cells play yet another role by storing the chemicals and thus effectively diluting levels in the blood, says Brigham and Women’s Hospital physician and Harvard environmental epidemiologist Susan Korrick. “There’s always the possibility that BMI [body mass index] or relative body fat could confound or mediate the relationship between exposures and outcomes,” says Korrick, who was not involved in the study.

Indeed, when adjusting for BMI, the authors of the current study found that many of the associations between higher exposures and later pubertal onset were weaker. In the case of PCBs, the relationship was reversed altogether—among girls with higher BMI, puberty occurred earlier, not later, with higher exposure.^5^

Recent studies on phthalates^14^ and phenols^15^ coauthored by Windham and also conducted within the Breast Cancer and the Environment Research Program found higher exposures were associated with both earlier and later pubertal development, depending on the chemical congener. “I don’t think there’s going to be one explanation for this phenomenon of earlier puberty, and there may still be other chemicals found associated,” Windham says.

In any case, the results of the new study don’t let the target compounds off the hook. “The chemicals are potentially affecting the endocrine system, related to delays in pubertal onset,” Windham says. “That might, in turn, influence pregnancy or other reproductive outcomes in the future. If it’s messing with the endocrine system in some way, we’re going to be concerned.”
